# Ganglioneuroma of the retroperitoneum presenting as a pediatric renal mass

**DOI:** 10.4103/0971-5851.64256

**Published:** 2009

**Authors:** Sanju Cyriac, Lakshmi Srinivas, N. Kathiresan, Shirley Sundersingh, Vandana Mahajan, T. G. Sagar

**Affiliations:** *Departments of Medical Oncology, Cancer Institute (WIA), Chennai, India*; 1 *Departments of Surgical Oncology, Cancer Institute (WIA), Chennai, India*; 2 *Departments of Pathology, Cancer Institute (WIA), Chennai, India*; 3 *Departments of Radiodiagnosis, Cancer Institute (WIA), Chennai, India*

**Keywords:** *Ganglioneuroma*, *pediatric*, *Wilm’s tumor*

## Abstract

Ganglioneuroma is the benign representative of peripheral neuroblastic tumors, with localized and predominant thoracic presentations in older children. They often have an excellent outcome with surgery alone. A 12-year-old girl presented with an incidentally detected abdominal mass, which was clinicoradiologically a renal mass. Laparotomy revealed a mass adherent to the anterior surface of the right kidney. The mass was carefully dissected out, sacrificing a portion of the inferior vena cava. Histopathological diagnosis was that of a ganglioneuroma. She was kept under follow up.

## INTRODUCTION

Ganglioneuroma (GN) is the benign counterpart in the spectrum of peripheral neuroblastic tumors (PNT).[1,2] It may arise *de novo* or from a neuroblastoma either spontaneously or after chemotherapy. A GN arising *de novo* usually affects older children, presents predominantly in the thorax, and has a variable amount of catecholamine secretion.[3] Characteristically, the localized disease is seen. The prognosis is excellent, as the surgical excision is often curative. Chemotherapy and radiotherapy have little role in its management.

We present the case of a 12-year old-girl who presented clinically with a right renal mass. Laparotomy and excision of the mass revealed a GN of the retroperitoneum.

## CASE REPORT

A 12-year-old girl presented to us with an incidentally detected abdominal mass. She was asymptomatic for any pain, altered bowel habits, fever or hematuria. The physical examination was unrevealing except for a right renal mass. Blood pressure was within normal limits.

**Figure 1:**
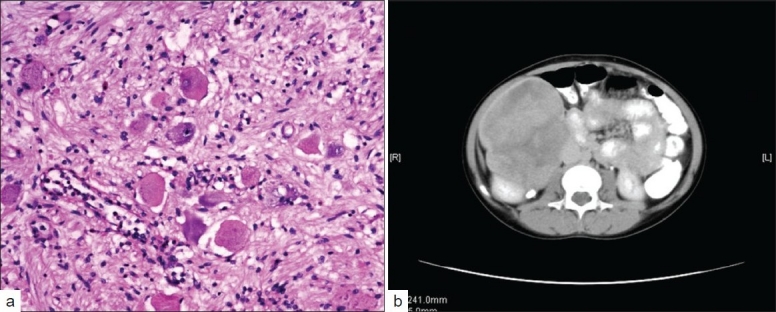
(a) Spindle shaped cells intermixed with mature ganglion cells (b) A large heterogenously enhancing mass apparently arising from the interpolar region of the right kidney extending towards the lower pole

Laboratory investigations revealed a normal hemogram, renal, and liver functions. She had normal 24-hour urine vanillylmandelic acid (VMA) levels and bilateral bone marrow studies. Furthermore, she was investigated with a CT scan of the abdomen [Figure 1a], which showed a large, heterogeneously enhancing mass lesion, arising from the interpolar region of the right kidney extending up to the under surface of the liver superiorly and medially, up to the IVC and laterally up to the abdominal wall. The mass was encasing the renal vessels, but patency was maintained. Nodes were not demonstrable.

She was taken up for laporatomy. Intraoperative findings revealed a retroperitoneal mass with nodular projections, densely adherent to the anterior surface of the right kidney, encasing the right renal vessels and extending to the IVC. The mass was carefully and completely dissected out with a 2.5 cm portion of the IVC, which was repaired. The histopathological examination was suggestive of GN [Figure 1b].

Final diagnosis was GN of the retroperitoneum. The patient was kept under follow up.

## DISCUSSION

Neuroblastoma, Ganglioneuroblastoma (GNB), and GN represent Peripheral Neuroblastic tumors (PNT) with a spectrum of neuronal maturation.[1] Ganglioneuroma can arise *de novo* or as a result of maturation of a neuroblastoma / Ganglioneuroblastoma, either spontaneously or secondary to treatment of a neuroblastoma. Ganglioneuroma, according to International Neuroblastoma Pathology Classification INPC criteria, is composed predominantly of ganglioneuromatous stroma with a minor component of scattered, evenly or unevenly distributed collections of differentiating neuroblasts and / or maturing / mature ganglion cells.[2] Hence, a complete excision and proper histopathological examination of the entire specimen is required, to rule out foci of immature neuroblasts as has been carried out in the index case.[1]

The clinical features depend on the site of the GN and the compression of the adjacent structures, although the majority are asymptomatic. They form a heterogenous group characterized by a predominant thoracic location, female preponderance, and are usually localized disease at presentation. Although lymph node metastases have been reported, many are due to primary GNB or neuroblastoma.[4] Also, adrenal, retroperitoneal (as seen in this case), pelvic, intestinal, bone, and neck lesions have been reported in literature.[1,4–6] Late malignant transformation of a benign lesion to neuroblastoma has also been reported.[7]

Typically, they are hypodense on CT with moderate contrast enhancement. Calcification may appear in a speckled pattern as previously described.[8] MRI may give a better assessment of the tumor extension than a CT scan.[9] Bruno *et al*. shared their experience with a retrospective study of 144 cases of GN that had excellent prognosis.[1] Seventy percent of their patients underwent complete resection and there was no recurrence recorded on follow up. Postoperative complications, about 18%, including two deaths were recorded.

Our patient was clinically and radiologically diagnosed as a case of renal mass with Wilm’s tumor as the closest differential. Intraoperative and histopathological analysis, however, confirmed GN (INPC criteria). Wilm’s tumor typically manifests as a solid intrarenal mass with a pseudocapsule, distorted renal parenchyma, and collecting system. It may displace or invade the adjacent vessels, but encasing is not seen typically.[10] A CT scan demonstrates a heterogenous mass and nodal metastases with areas of calcification. Renal cell carcinoma (RCC) is usually smaller and has a more heterogenous enhancement and calcification compared to Wilm’s tumor. Other rarer tumors have characteristic imaging features such as involvement of renal sinus in a mesoblastic nephroma or multiple cysts with variably enhancing septa in a multi locular cystic renal tumor. The age, encasement of renal vessels by the tumor without lumen compromise, and histopathological findings that were typical features of GN, influenced surgical excision. Aggressive surgery is not recommended because the complication rate is quite high, but efforts should be made to debulk the tumor as much as possible.[1]

In conclusion, this is a case of histopathologically confirmed retroperitoneal GN that clinically and radiologically mimicked an intrarenal mass in a 12-year-old girl. She has enjoyed stable health following surgical excision, which is usually curative. A high index of suspicion is required to diagnose this lesion.
